# Ice VII from aqueous salt solutions: From a glass to a crystal with broken H-bonds

**DOI:** 10.1038/srep32040

**Published:** 2016-08-26

**Authors:** S. Klotz, K. Komatsu, F. Pietrucci, H. Kagi, A.-A. Ludl, S. Machida, T. Hattori, A. Sano-Furukawa, L. E. Bove

**Affiliations:** 1 Institut de Minéralogie, de Physique des Matériaux et de Cosmochimie, CNRS UMR 7590, Université Pierre-et-Marie-Curie, F-75252 Paris, France; 2 Geochemical Research Center, Graduate School of Science, The University of Tokyo, Tokyo 113-0033, Japan; 3 CROSS-Tokai, Research Centre for Neutron Science and Technology, 162-1 Shirakata, Tokai, Ibaraki 319-1106, Japan; 4 J-PARC Center, Japan Atomic Energy Agency, Tokai, Naka, Ibaraki 319-1195, Japan; 5 Institute of Condensed Matter Physics, Ecole Polytechnique Fédérale de Lausanne, CH-1015 Lausanne, Switzerland

## Abstract

It has been known for decades that certain aqueous salt solutions of LiCl and LiBr readily form glasses when cooled to below ≈160 K. This fact has recently been exploited to produce a « salty » high-pressure ice form: When the glass is compressed at low temperatures to pressures higher than 4 GPa and subsequently warmed, it crystallizes into ice VII with the ionic species trapped inside the ice lattice. Here we report the extreme limit of salt incorporation into ice VII, using high pressure neutron diffraction and molecular dynamics simulations. We show that high-pressure crystallisation of aqueous solutions of LiCl∙RH_2_O and LiBr∙RH_2_O with R = 5.6 leads to solids with strongly expanded volume, a destruction of the hydrogen-bond network with an isotropic distribution of water-dipole moments, as well as a crystal-to-amorphous transition on decompression. This highly unusual behaviour constitutes an interesting pathway from a glass to a crystal where translational periodicity is restored but the rotational degrees of freedom remaining completely random.

All forms of ice are made of hydrogen-bonded networks of H_2_O molecules with local tetrahedral coordination, see refs 1, 2, 3, 4 and references therein. Such geometry is thought to be incompatible with the presence of ionic species since the electric field of an ion interacts strongly with the electric dipole moment of the water molecule and perturbs the directional H-bonding to its neighbours^5^. Indeed, when salt water freezes it tends to expel the salt, as observed in the shelf ice of freezing sea water^6^. A related effect is the ‘salting-out’ of proteins by concentrated salt solutions^5^. In general, the interaction of water with dissolved ionic species is of fundamental interest for various fields of research ranging from life sciences to planetology. High-pressure ice in contact with salt is a major ingredient in the geology of satellites in the outer solar system^7^,^8^ and the understanding of the interior of these bodies rely critically on our knowledge of properties of aqueous salt solutions under strong compression.

Recently, it has been shown that the most stable high-pressure ice phase, ice VII, can accommodate significant amounts of Li^+^ and Cl^−^ ions, if prepared by pressure-induced crystallisation of the glassy solution^9^. In fact, in a narrow concentration range between R = 5.5 and R = 6.5, aqueous LiCl and LiBr solutions LiCl∙RH_2_O and LiBr∙RH_2_O easily form glasses, simply by cooling the liquid, see Fig. 1 10,11. When such a solid solution with R = 6 is compressed below 100 K and heated, it crystallises into ‘salty ice VII’ where the Li-ions are trapped interstitially in the octahedral voids of the bcc-type ice VII structure, and the Cl^−^ ion are located at water sites^9^. Such ‘doping’ of ice VII leads to an increased unit cell volume and a local perturbation of the hydrogen bond network which is visible in the diffraction patterns by an increased Debye-Waller factor. The very narrow range wherein such solid amorphous aqueous solutions can be prepared (Fig. 1) suggests that the crystallisation behaviour into ice VII depends sensitively on the salt concentration. In the following we show that this is indeed the case, i.e. that the structure of such crystallized salty ice VII is highly sensitive to the amount of salt inclusions, and that they give rise to salty ices with strongly expanded volumes, random water-dipole orientation and crystal-amorphous transitions under decompression at low temperatures.

For this purpose, deuterated aqueous solutions with R = 5.6 of LiCl and LiBr (LiCl∙5.6D_2_O, and LiBr∙5.6D_2_O) were prepared. They present the limit of salt concentration where a homogeneous glass can easily form without applying extreme cooling speeds. We used deuterated samples to avoid the very large incoherent scattering of hydrogen, a standard method applied in powder neutron diffraction, including in almost all previous structural work on ice^2^,^3^,^4^,^9^ (the isotope effect on the structure and phase diagram of ice is known to be negligible). The vitrified samples were then compressed at 80–85 K to pressures between 4 and 6 GPa and warmed to 300 K while keeping the pressure approximately constant. Neutron diffraction patterns were collected during the warm-up with accumulation times between 5 and 20 minutes. Figure 2 shows the behaviour of both the LiCl- and the LiBr-samples close to room temperature. Crystallisation of the glass is observed to start at ≈270 K and reaches a stable state slightly above 300 K. The diffraction patterns are characterized by a single, large peak at d ≈ 2.5 Å corresponding to the 110 Bragg reflection of ice VII. All other (sharp) reflections are either from a small amount of lead which was added as a pressure marker, or tungsten carbide which is the anvil material. Obviously, the high-pressure crystallized solids are far from being perfect crystals, but they certainly cannot be qualified as a glass either. In fact, the patterns are sufficiently crystalline to be able to analyze them by Rietveld methods, as shown in Fig. 3 for the two systems. Without surprise the fits are not perfect since they cannot reproduce the significant diffuse scattering which leads to the asymmetric wings around the 110 reflection. The large width of the reflection is most likely a particle size effect (typical size: 20 Å), and to a minor extent due to strain, as deduced from the refined peak shape parameters.

The most surprising feature of these patterns is, however, the total absence of the 111 reflection which should occur at ≈2.0 Å (red arrows in Fig. 3), and which is clearly present in samples with lower salt concentrations^9^. This cannot be an effect of the strong Debye-Waller factor since the 211 reflection at lower d-spacing (≈1.35 Å, blue arrows in Fig. 3) is clearly visible, though weak, as expected. In the ice VII structure, the 111 reflection is *entirely* due to hydrogen (deuterium), i.e. it has no contribution from oxygen. Therefore, its absence is a clear and unambiguous proof of hydrogen being strongly displaced from its normal position along <x,x,x>, see Fig. 5 for a sketch of the ice VII structure and inset of Fig. 3 for a comparison with a pattern of pure ice VII. In initial fits this effect was simulated by varying displacement factors for hydrogen which gave <x^2^>^1/2^ ≈ 0.6 Å, which is indeed large. For comparison, the displacements in pure ice VII at 2.6 GPa and 295 K are <x^2^>^1/2^ ≈ 0.15–0.20 Å^12^,^13^, i.e. more than three times smaller. A more realistic model with 8 positions was hence adopted, 4 positions along <x,x,x>, and 4 others at the anti-tetrahedral (‘interstitial’) positions, a geometry which describes a more isotropic distribution of H around O. Refinements in this model gave better fits with reduced H-displacements of <x^2^>^1/2^ ≈ 0.30 Å. Fits based on this model correspond to the lines through the data shown in Fig. 3, and results of the refinements are given in Table 1. In conclusion, whatever the detail of the model adopted, the evidence is that hydrogen is more or less isotropically disordered. This necessarily entails – given the fact that the molecular geometry is intact - that the electric dipole moment has no preferred orientation with respect to the crystallographic axes.

The second remarkable feature is the strongly expanded lattice parameter: at the pressures measured by the lead marker the volume expansion relative to pure ice VII is 14% and 18% for the LiCl- and LiBr-system, respectively. These values scale *exactly* with the difference in size r of the anions involved, i.e. r_Cl_ = 1.81 Å and r_Br_ = 1.96 Å, i.e. it is an unambiguous proof that the ions are indeed included into the structure, on specific lattice sites. Surprisingly, the volume expansion is not proportional to the salt content: For a 7% lower LiCl concentration (R = 6), the volume expansion was observed to be 43% smaller (i.e. only 8%)^9^. This sensitivity to the salt concentration must be related to the fact that – contrary to the low pressure ice forms - phase VII achieves its high density through interpenetration, i.e. by forming two interpenetrating but non-connected sublattices. Such a configuration permits, for example, to have the shortest O-O distances larger than in ordinary ice I_h_, despite its considerably larger density. The destruction of the interpenetrating sublattices by the presence of ionic species must necessarily lead to an increase of volume which is expected to be highly sensitive to the ion concentration.

The structural properties of these highly salt-loaded ice phases were then investigated by molecular dynamics simulations which give access to physical properties which cannot be extracted by diffraction measurements alone. For this purpose simulations were carried out with 15 × 15 × 15 ice VII supercells corresponding to 19227 atoms. Note that the structural results are insensitive to the choice of the isotope (hydrogen or deuterium) since the potentials used in the simulations are the same. Details of the calculations are described in the Methods section.

Figure 4 shows a snapshot of typical molecular configurations in LiCl∙5.6H_2_O compared to pure ice VII, viewed along the cubic [100] direction. Results for the LiBr-system are qualitatively similar and are not shown here. It is seen that inclusion of Li^+^ and Cl^−^ leads to molecular displacements of several tenths of angstroms, but *on average* the H_2_O molecules are still at the bcc sites of ice VII. It is also seen that the Li^+^-ions prefer to locate themselves in the octahedral interstitials, whereas the much larger Cl-ions are on average closer to sites where H_2_O is expected. Radial distribution functions were calculated for the two systems and can be found as Supplementary Information. These indicate an average of three H-atoms up to a distance of 2.1 Å compared to four in pure ice VII, suggesting that a sizeable number of H-bonds are broken.

The most important detail in this context concerns however the spatial distribution of dipole moments. For this purpose the orientation of each water molecule was extracted from the last part of the 12 ns trajectory (1 ns) and employed to build the spherical probability maps shown in Fig. 5. The probability density is normalized so as to give an integral over all orientations equal to one. For comparison, a similar-size model of ice VII was also built and equilibrated at constant pressure P = 10 GPa and T = 300 K (see Methods for the choice of the pressure).

As shown in the Fig. 5, pure ice VII is characterized by molecular dipoles sharply aligned along [100] and equivalent directions, as expected. In LiCl∙5.6H_2_O and LiBr∙5.6H_2_O, however, the orientational probability is almost exactly isotropic. Close inspection of the dipole distribution shows a small residual preference for <100> directions in the case of LiCl∙5.6H_2_O which is hardly discernible in Fig. 5, but an almost isotropic distribution for the Br-system. This is confirmed by calculating the standard deviation σ of the probability over all dipole orientations. For the Cl-system we find σ(LiCl) = 0.012, for the Br-system σ(LiBr) = 0.0090, with average probabilities of 0.075 (LiCl) and 0.078 (LiBr), i.e. relative variations of only 16% and 11%, respectively.

The simulations therefore come to the same conclusion as the diffraction data: The ice VII-like structures emerging from crystallisation of highly concentrated LiCl and LiBr-solutions under pressure have completely disordered molecular orientations which means that a large fraction of hydrogen-bonds are broken and that the ice-rules are violated. This distinguishes it sharply from the orientational disorder present in most of the (pure) ice phases where the ice-rules are conserved. The crystallisation observed here could be classified as ‘fractional’ since it concerns mainly the three translational degrees of freedoms whereas the three rotational freedoms remain uniformly random. Breaking of hydrogen bond networks is a phenomenon which has been observed in liquid salt solutions at ambient pressure^10^,^14^, and also in pure liquid water above the critical point^15^. For solid water, however, including amorphous ice phases, this seems to be a new observation which is only possible by crystallisation of the solutions under pressure.

In this context the question arises if the inclusion of ionic species into ice VII promotes “plasticity”, i.e. the formation of an ice VII phase with freely rotating molecules, as predicted by several simulations^16^,^17^,^18^ to occur close to the melting line. A diffraction experiment can – by principle – not give information on the dynamics of molecular orientations, but our simulations can safely exclude this scenario. In fact, the calculated orientational correlation times of water dipoles in LiCl∙5.6H_2_O and LiBr∙5.6H_2_O are typically 4–5 times longer compared to pure ice VII at the same pressure. Therefore, although the ions severely perturb the H-bond network, their presence seems to hamper their orientational dynamics rather than to promote it.

A further interesting aspect concerns the behaviour under de-compression at low temperatures: The two salty ice-forms are found to be *not* quenchable at 90 K, and probably even not at any temperature below, contrary to all forms of pure ice. Figure 6 shows diffraction patterns of the two systems at ambient pressure and the same temperature, before compression and after the compression/crystallisation cycle, after decompression at ≈90 K. The surprising finding is that both ice VII forms of LiCl∙5.6D_2_O and LiBr∙5.6D_2_O return to an amorphous solid, a disordered system which is *significantly different* from the original glass. A comparison with published structure factors from ref. 19 shows a striking resemblance with high density amorphous ice (HDA), except that the main diffraction feature is at ≈0.2 Å shorter d-spacing, approximately at a position where it is found in very high density amorphous ice (VHDA)^20^. From this we suspect that upon decompression, the hydrogen-bond network might be partially restored with a slightly higher molecular density than found in HDA. Hence, we observe a strong memory effect: the vitrified glass has a memory of the liquid with its structure governed by hydration of the ionic species, whereas the de-compressed amorphous ice has kept a memory of the crystalline ice VII, where the cohesion is governed by the need for maximizing the packing density.

In conclusion, we have presented neutron diffraction and molecular simulation results on highly concentrated LiCl- and LiBr aqueous solutions which were crystallized under high pressure. They indicate that the ionic species can be trapped inside a strongly expanded ice VII – like structure with random electric dipole orientation. These structures cannot be quenched (‘recovered’) to ambient pressure and low temperatures but transform back to a glass with a structure different to the initial one. The trapping of ionic species by crystallisation from a glass (devitrification) might be found in other network forming glasses, for example in silica. In fact, “stuffed” SiO_2_ polymorphs incorporating small ions such as lithium are well known and can be formed at ambient pressure^21^. The high-pressure route used in this study might enable to synthesize “stuffed” high pressure silica phases which are expected to have remarkable properties, for example improved hardness, and therefore might be of technological interest.

## Methods

### Sample preparation

Aqueous solutions of LiCl and LiBr with R = 5.6 were prepared from the salts purchased from Aldrich, and 99.8% enriched heavy water (D_2_O) from Eurisotop, France. The solutions were filtered to remove particles larger than 3 μm to minimize the possibility of crystallisation during cooling, and loaded into TiZr gaskets together with a small piece of lead (10 mg). After sealing the gasket with a load of 10 kN onto the anvils the cell was cooled at a speed of 10 K/min to 80 K which transformed the liquids into pure glasses. The samples were then compressed at 80–110 K in steps up to 4–5 GPa and then heated at an average speed of typically 1 K/min up to the temperature where crystallisation occurred. After crystallisation, the samples were stable over at least the timescale of the diffraction experiments, i.e. 1–3 hours. Samples at lower pressures (below ca. 3.3 GPa for the LiCl compound and ca. 4.3 GPa for the LiBr system) crystallized into solids with unknown structure.

### Neutron diffraction

High pressure neutron diffraction measurements were carried out at the Japanese high pressure beamline PLANET^22^ at MLF, the Japan Accelerator Research Complex (J-PARC), Tokai, Ibaraki, Japan, using a variable P-T hydraulic press (‘Mito system’)^23^, and tungsten carbide anvils with a profile as described in ref. 24 and 9/6 mm outer/inner diameters. Encapsulating gaskets were made of null-scattering TiZr, surrounded by a supporting ring of a high-tensile aluminium alloy^24^. Normalisation of the data was achieved in a separate run using a vanadium pellet and an anvil configuration which was strictly similar.

### Molecular dynamics simulations

Salty ices of stoichiometry Li(Cl/Br)∙5.6H_2_O have been simulated employing the TIP4P/Ew interatomic potential for water^25^ and the potentials of Joung & Cheatham for Li^+^, Cl^−^, and Br^−^ ions^26^. The potentials for hydrogen and deuterium are strictly identical which allows us to make meaningful comparison with the neutron diffraction data which used deuterated samples. Molecular dynamics simulations were performed employing the software Gromacs^27^. The temperature was controlled with the stochastic velocity rescaling thermostat^28^, and the pressure with a Berendsen barostat^29^. For each composition (Cl or Br) samples were generated in the following way: Ice VII 15 × 15 × 15 supercells with 19227 atoms were constructed employing the experimental lattice parameter (a = 3.42835 Å with Cl and a = 3.45795 Å with Br) and positioning 6750 water molecules at ideal bcc lattice sites with a random orientation of dipoles, imposing the ice rules. 1023 water molecules, selected at random, were removed and replaced with either Cl^−^ or Br^−^ ions. Finally, 1023 Li^+^ ions were introduced choosing randomly their positions among the centers of octahedra defined by water molecules and halide ions. These initial geometries were constructed avoiding to place two identical ions closer than the lattice parameter a. The composition corresponds to a stoichiometry Li(Cl/Br)∙5.63H_2_O. Each structure was initially relaxed at T = 10 K and constant volume for 30 ps (with a timestep of 0.1 fs). Then, the system was slowly heated up to T = 300 K employing a time constant of 50 ps for the thermostat (with a timestep of 1 fs) and equilibrated for 12 ns. The dipole moment orientation of each water molecule was extracted from the last part of the trajectory (1 ns) and employed to build the spherical probability maps. The orientational correlation time of water dipoles was estimated performing, for each system, additional 50 ns-long simulations using deuterium instead of hydrogen masses. In a second set of simulations a smaller 4 × 5 × 7 cell was used to generate 100 configurations which served to calculate neutron diffraction patterns and verify the consistency with experiment. Note that constraining the system at the experimental volume corresponds to a pressure of about 10 GPa: this is consistent with the domain of stability of ice VII with the interatomic potential employed^30^, whereas imposing the experimental pressure (4–5 GPa) to the theoretical samples would correspond to the stability domain of the liquid resulting in much more disordered structures.

## Additional Information

**How to cite this article**: Klotz, S. *et al*. Ice VII from aqueous salt solutions: From a glass to a crystal with broken H-bonds. *Sci. Rep.* **6**, 32040; doi: 10.1038/srep32040 (2016).

## Supplementary Material

Supplementary Information

## Figures and Tables

**Figure 1:**
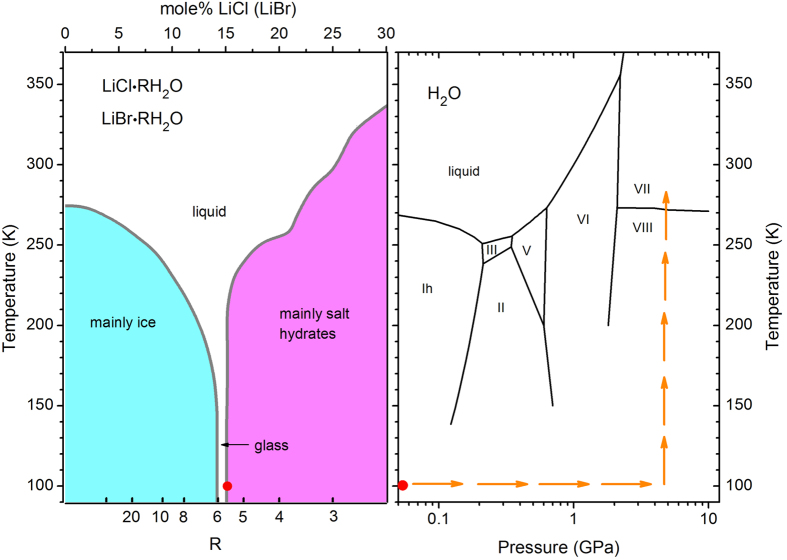
Left panel: Schematic metastable phase diagram of LiCl∙RH_2_O and LiBr∙RH_2_O, redrawn after refs ****10**^10^,^11^**. In a small region of concentration R ≈ 5.5–6, the supercooled liquid transforms directly into a glass. Regions to the left and the right are stability domains of ice and various hydrates. Right panel: Pressure-temperature path applied to form LiCl- and LiBr containing ice VII together with the phase diagram of pure water. Red dots indicate initial sample conditions at ambient pressure.

**Figure 2:**
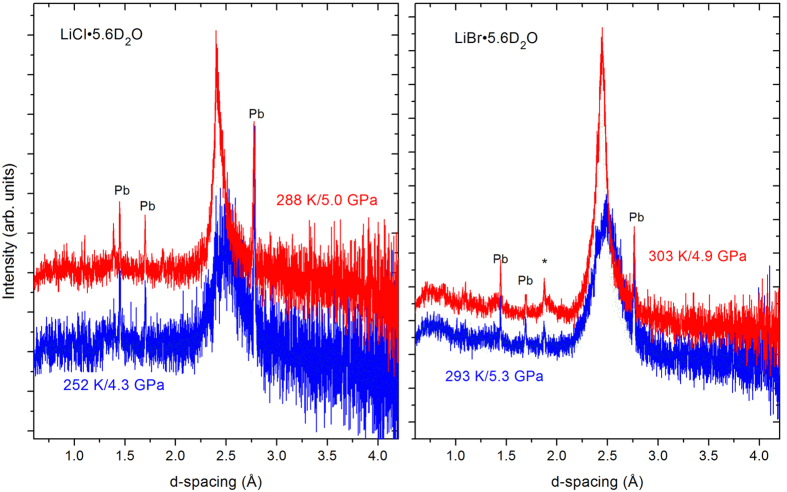
Neutron diffraction patterns taken during crystallisation of compressed LiCl∙5.6D_2_O (left, accumulation time 5 min) and LiBr∙5.6D_2_O (right, accumulation time 10 min) upon warming. Sharp reflections are of a small quantity of lead (Pb) mixed with the sample which serves as pressure marker, the asterisk is a reflection of the tungsten carbide anvils. The high temperature patterns were shifted vertically to avoid overlap.

**Figure 3:**
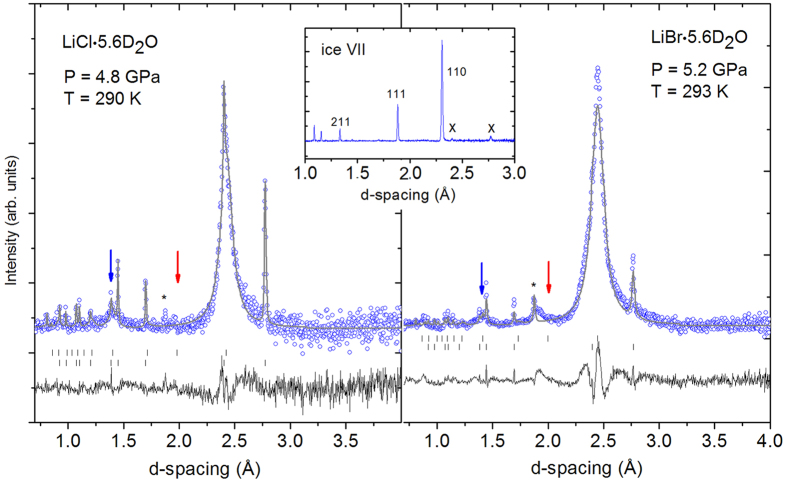
Neutron diffraction patterns (circles) and Rietveld fits (lines) to the data using the model described in the text. Sharp peaks are from lead, the asterisk is a reflection from the tungsten carbide anvils. Expected positions of 111 and 211 reflections are indicated by red and blue arrows, respectively. Upper and lower tick marks correspond to reflections of ice VII and lead, respectively, the difference curves are shown below. Accumulation time is 15 (left) and 30 minutes (right). For comparison, a pattern of well-crystallised pure ice VII at 4.7 GPa is shown in the inset including Miller indices of the three strongest reflections. Crosses indicate reflections of the lead pressure marker.

**Figure 4:**
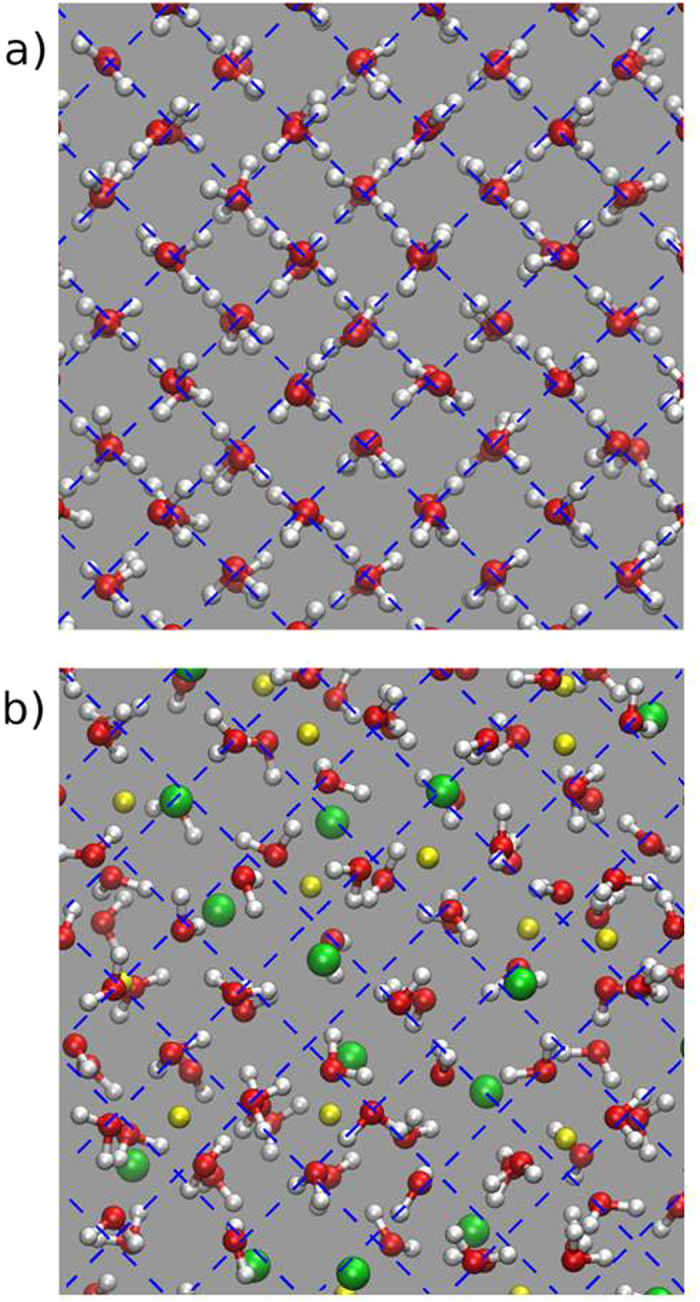
Snapshots of molecular configuration in pure ice VII (**a**) and LiCl∙5.6H_2_O (**b**), viewed along the cubic [100] direction. Li-ions are drawn in yellow, Cl-ions in green. The dashed lines are guides to the eye.

**Figure 5:**
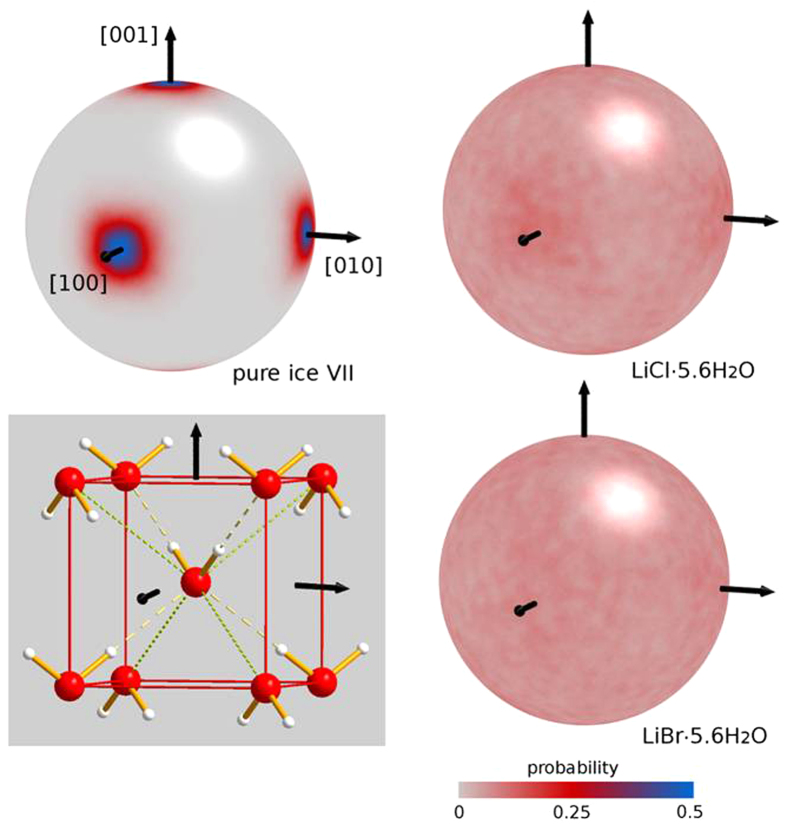
Probability density distribution of the electric dipole moment of H_2_O in pure ice VII (upper left), LiCl∙5.6H_2_O (upper right) and LiBr∙5.6H_2_O (lower right), derived from molecular dynamics simulations. The distribution was normalized to give an integrated probability equal to one. Note a very weak preference of dipoles along <100> in the LiCl-system, and a completely flat distribution for the LiBr-system. A sketch of a typical instantaneous configuration in pure ice VII is given (lower left) with hydrogen bonds indicated as dashed lines and the non-bonding (“anti-tetrahedral”) directions as dotted lines.

**Figure 6:**
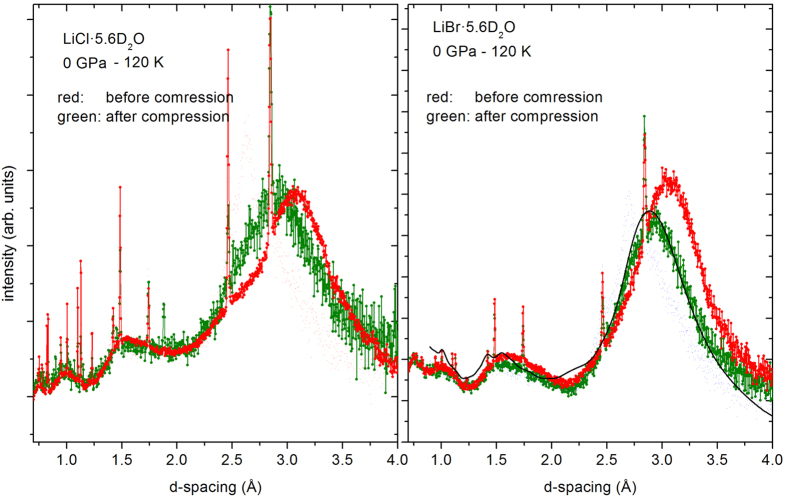
Neutron diffraction patterns at ambient pressure, before compression of the glass, and after decompression of the crystallized phases (see Fig. 3). For comparison, the solid line represents neutron data for high density amorphous ice (HDA) from ref. 19.

**Table 1:** Structural parameters derived from Rietveld refinements to neutron diffraction patterns shown in Fig. 3.

	LiCl∙5.6D_2_O	LiBr∙5.6D_2_O
Pressure (GPa)	4.8(1)	5.2(1)
a (Å)	3.4284(17)	3.4580(20)
ΔV/V	14%	18%
<x^2^>^1/2^ (Å) D/O	0.31/0.18	0.50/0.25

Data were refined in pace group 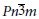 of ice VII^12^,^13^ with oxygen at (0.25, 0.25, 0.25), hydrogen (deuterium) at the 4 tetrahedral (0.41, 0.41, 0.41) positions as well as 4 further “anti-tetrahedral” sites at (0.08, 0.08, 0.08). Li was placed at interstitial octahedral sites at (0.75, 0.75, 0.25) and Cl (Br) at O-sites. Pressure values were determined from the refined lattice parameters of lead which was added to the sample^31^. a is the refined lattice parameter of the samples, ΔV/V the volume expansion compared the pure ice VII, using the equation of state of ref. 32, <x^2^>^1/2^ is the mean square displacement of deuterium/oxygen.
