# Supplementary material for: Ice VII from aqueous salt solutions: From a glass to a crystal with broken H-bonds
Source: Sci Rep. 2016 Aug 26;6:32040. doi: 10.1038/srep32040 (PMC5000010; doi:10.1038/srep32040)
Supplement: Supplementary Information [file srep32040-s1.pdf]

Supplementary Information

Ice VII from aqueous salt solutions: From a glass to a crystal with broken H-bonds

S. Klotz[1,*], K. Komatsu[2], F. Pietrucci[1], H. Kagi[2], A.A. Ludl[1], S. Machida[3], T. Hattori[4], A. Sano-Furukawa[4], L.E. Bove[1,5]

[1]Institut de Minéralogie, de Physique des Matériaux et de Cosmochimie, CNRS UMR 7590, Université Pierre-et-Marie-Curie, F-75252 Paris, France.

[2]Geochemical Research Center, Graduate School of Science, The University of Tokyo, Tokyo 113-0033, Japan.

[3]CROSS-Tokai, Research Centre for Neutron Science and Technology, 162-1 Shirakata, Tokai, Ibaraki 319-1106, Japan.

[4]J-PARC Center, Japan Atomic Energy Agency, Tokai, Naka, Ibaraki 319-1195, Japan.

[5]Institute of Condensed Matter Physics, Ecole Polytechnique Fédérale de Lausanne, CH-1015 Lausanne, Switzerland.

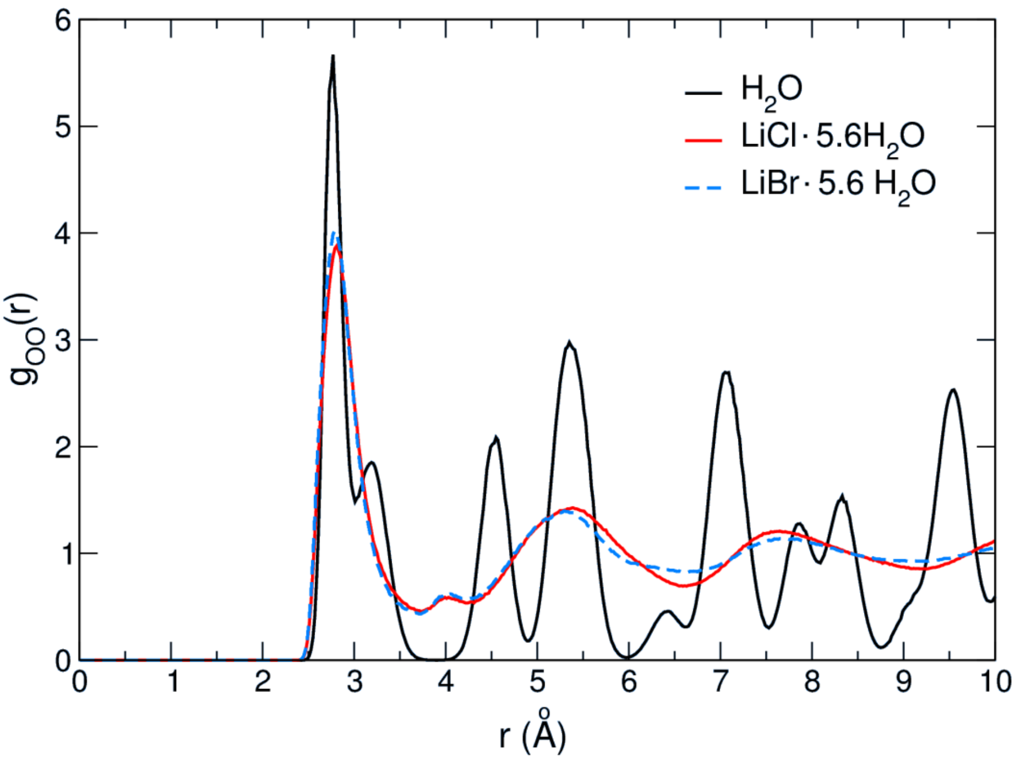

Fig. S1 : Oxygen-oxygen radial distribution function of $LiCl{\cdot}5.6H_2O$ (red) and $LiBr{\cdot}5.6H_2O$ (blue) in comparison with pure ice VII (black), from molecular simulations. Peak positions in pure ice VII correspond to the nearest neighbour hydrogen-bonded distances along [111] at 2.8 Å, the next-nearest neighbour distance along [100] (3.15 Å), the third neighbour distance along [110] at 4.5 Å, and the fourth neighbour distance along the [111] body diagonal.

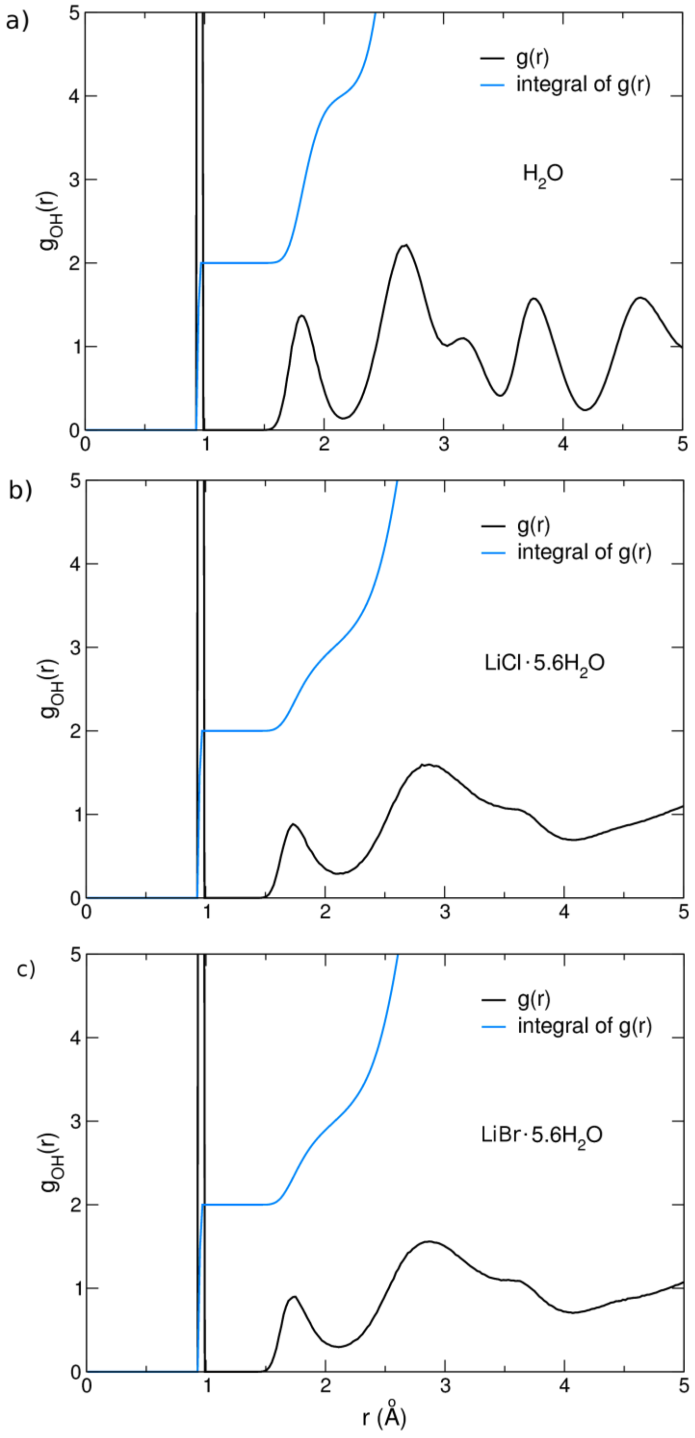

Fig. S2 : Oxygen-hydrogen radial distribution function (black) and integral corresponding to H-coordination number in $LiCl\cdot5.6H_2O$ (b), $LiBr\cdot5.6H_2O$ (c), and pure ice VII (a), from molecular simulations. The sharp peak in $g_{OH}(r)$ at 0.98 Å corresponds to the covalent O-H, the peak at 1.8 Å to the H-bonded hydrogen of the neighbouring water molecule.
